# The Effect of Progressive Muscle Relaxation Technique on Fatigue and Depression in Patients With Hypothyroidism: A Randomized Controlled Trial

**DOI:** 10.1002/hsr2.72893

**Published:** 2026-07-25

**Authors:** Mehdi Safarabadi, Marzieh Mousivand, Hooman Mohammad talebi, Hossein Poorcheraghi, Sara Mohammadi Farsani

**Affiliations:** ^1^ Department of Paramedicine Arak University of Medical Sciences Arak Iran; ^2^ Students Research Committee Khomein University of Medical Sciences Khomein Iran; ^3^ Faculty Member of Nursing Department Khomein University of Medical Sciences Khomein Iran; ^4^ Department of Community Health and Geriatric Nursing, School of Nursing and Midwifery Tehran University of Medical Sciences Tehran Iran

**Keywords:** depression, fatigue, hypothyroidism, muscle relaxation, nursing

## Abstract

**Background and Aims:**

This study aimed to evaluate the effectiveness of progressive muscle relaxation in reducing fatigue and depressive symptoms and improving quality of life among patients with hypothyroidism experiencing persistent symptoms despite standard thyroid hormone replacement therapy.

**Methods:**

This single blind randomized controlled trial included 68 adults with hypothyroidism in the Fateme‐Zahra Hospital, Khomain. Participants were randomly allocated to an intervention group receiving progressive muscle relaxation (PMR) based on the Jacobson relaxation method or a control group receiving routine care. The intervention group performed PMR twice daily for 4 weeks. Fatigue and depressive symptoms were assessed at baseline and post‐intervention using validated questionnaires. Ethical approval and written informed consent were obtained.

**Results:**

All 68 participants completed the study, and baseline demographic characteristics were largely comparable between groups. The control group showed no significant changes in fatigue or depression over 4 weeks. In contrast, the intervention group exhibited significant reductions in fatigue (mean change −41.45, *p* < 0.001) and depressive symptoms (mean change −8.09, *p* < 0.001). Between‐group comparisons demonstrated significantly greater improvements in the intervention group, with large effect sizes, indicating a substantial benefit of progressive muscle relaxation. Adjusted analyses confirmed significant intervention‐related reductions in fatigue and depression independent of baseline covariates and comorbidities.

**Conclusion:**

Progressive muscle relaxation, alongside educational and behavioral interventions, significantly reduced fatigue and depressive symptoms in patients with hypothyroidism. Further long‐term studies are warranted.

**Trial Registration:** (IRCT20250605066079N1) − 2025‐07‐04.

## 1 Introduction

Hypothyroidism is a common non‐communicable endocrine disorder characterized by insufficient secretion of thyroid hormones. Recent estimates suggest that thyroid disorders affected around 750 million individuals worldwide as early as 2012, and the number has likely increased since then [1, 2].

Recent population‐based research indicates that the prevalence of hypothyroidism in the general population ranges from approximately 0.3% to 3.7% in the United States and 0.2% to 5.3% in Europe, with a larger segment of adults (~12%) exhibiting subclinical hypothyroidism [2]. Global estimates suggest that more than 750 million people may have thyroid disorders, and the risk of developing hypothyroidism is 5–8 times higher in women than men [3]. On the other hand, hypothyroidism accounts for 1% and 0.9% of undiagnosed clinical and subclinical hypothyroidism, respectively [4, 5]. These figures underscore the substantial public health impact of hypothyroidism.

Despite its high prevalence, hypothyroidism often goes unrecognized because the onset is insidious and symptoms are nonspecific. Classic clinical manifestations include fatigue, weakness, weight gain, cold intolerance, and constipation [6]. Fatigue remains debilitating in treated hypothyroidism: a 2025 UK survey of 1251 patients reported a mean FACIT‐F score of 20.5 ± 10.5, with 89% showing abnormal fatigue despite thyroid hormone therapy [7]. Fatigue is a persistent symptom that impairs quality of life, often remaining despite normalized thyroid function, and is frequently accompanied by mood disorders. A 2021 meta‐analysis of 348,014 participants found hypothyroidism increased the odds of depression by 30% (OR 1.30), with stronger associations for overt hypothyroidism (OR 1.77) and women (OR 1.48) [1]. A cross‐sectional analysis of 12,502 U.S. adults found depressive symptoms were significantly inversely correlated with FT4, FT3, and TT3 in both younger (< 60 years) and older adults. A positive association with thyroid peroxidase antibodies (TPOAb) was observed only in younger adults [8]. These studies underscore that hypothyroidism is associated with both physical and psychological burden.

Many hypothyroidism patients continue to experience fatigue, depression, “brain fog,” and other symptoms despite biochemical euthyroidism [9, 10]. Residual symptoms have prompted interest in PMR, a safe, low‐cost technique that relaxes muscles and reduces stress. Residual fatigue and mood disturbances in hypothyroidism may result from autonomic imbalance, heightened sympathetic activity, and stress‐related neuroendocrine changes [11]. Progressive Muscle Relaxation (PMR) systematically tenses and relaxes muscles, reducing sympathetic arousal and enhancing parasympathetic tone [12]. This shift can lower cortisol, improve sleep, and reduce inflammation, thereby increasing energy and alleviating fatigue. Additionally, PMR may modulate central pathways involved in mood regulation, helping to reduce depressive symptoms [13]. High‐quality evidence suggests that PMR can ameliorate fatigue, depression, and sleep disturbances across diverse populations [14, 15]. A randomized controlled trial (RCT) in adults with cystic fibrosis incorporated PMR into pulmonary rehabilitation for 48 days and reported significant reductions in depression (Hospital Anxiety and Depression Scale‐Depression subscore) compared with standard rehabilitation (*p* = 0.02) [16]. Collectively, these studies demonstrate that PMR produces meaningful benefits in fatigue and mood across various clinical contexts. Despite adequate thyroid hormone replacement, many patients continue to experience fatigue and depressive symptoms [17]. While mind–body and behavioral interventions have shown benefits in other chronic conditions, controlled trials evaluating their efficacy in patients with treated hypothyroidism are lacking. This gap underscores the need for rigorously designed studies to assess whether interventions such as PMR can alleviate residual symptoms in this population [18].

Although PMR has shown promise in reducing fatigue and depression in various chronic illnesses, its effects in patients with hypothyroidism remain unclear. Given the high prevalence of residual symptoms in treated hypothyroidism and the burden these symptoms impose on patients' quality of life, investigating non‐pharmacologic interventions is clinically relevant. The present study therefore aims to evaluate the effectiveness of progressive muscle relaxation in reducing fatigue and depressive symptoms and improving quality of life among patients with hypothyroidism.

## 2 Methods

In this randomized controlled trial, 68 patients diagnosed with hypothyroidism (based on the physician's diagnosis) who visited the Fatima Zahra Hospital clinic in Khomain and provided written informed consent to participate in the intervention were included in the study. Participants were randomly assigned to either the intervention group (A) or the control group (B) using a computer‐generated randomization sequence created in Microsoft Excel 2019 by an independent researcher not involved in recruitment or assessment. Participant enrollment was conducted by the principal investigator, while group allocation was concealed using sequentially numbered, sealed opaque envelopes until assignment. The study was ethically approved by the Khomain Medical Sciences University Ethics Committee.

**Figure 1:**
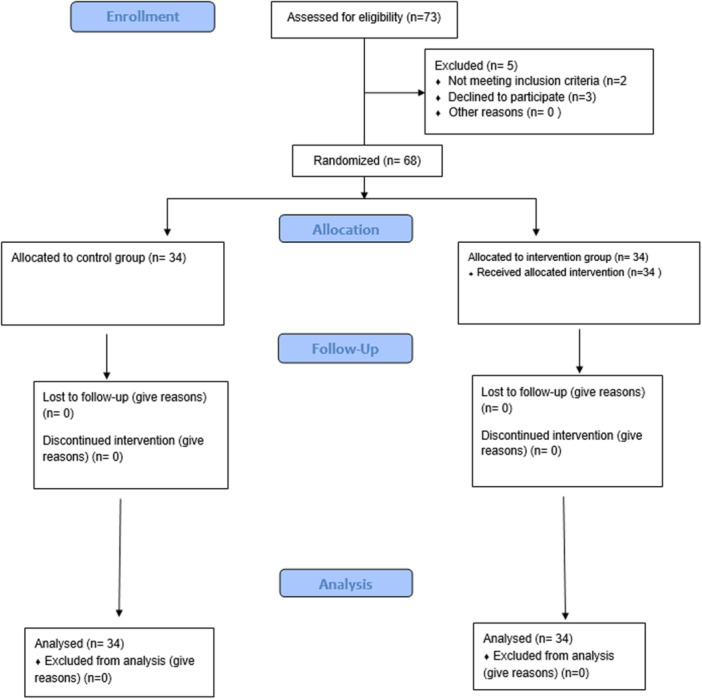
CONSORT diagram of participant allocation.

Before the intervention, all participants completed a set of questionnaires, including a demographic information form, the Piper Fatigue Scale, the Beck Depression Inventory (BDI‐2). In the first session, both groups received information about the disease and its complications. Then, the Progressive Muscle Relaxation Technique (PMRT) was taught to the intervention group (A) by the researcher in a separate room for 40 min. The study followed a single‐blind design, meaning that the researcher who assessed the outcomes and the data analyst were blinded to the group allocation. Due to the nature of the intervention, participants were aware of whether they received Progressive Muscle Relaxation Training (PMRT). Figure 1 shows participant allocation process.

### 2.1 Diagnostic Criteria for Hypothyroidism

To improve diagnostic consistency and reproducibility, hypothyroidism was defined based on an endocrinologist‐confirmed diagnosis and laboratory findings documented in the patients' medical records. Eligible participants had elevated serum thyroid‐stimulating hormone (TSH) levels with decreased free thyroxine (FT4) levels consistent with overt primary hypothyroidism, or elevated TSH levels with normal FT4 levels consistent with subclinical hypothyroidism according to standard clinical criteria [19, 20]. Participants were required to have a stable clinical condition and to be receiving levothyroxine treatment for at least 3 months prior to enrollment, with no dose adjustment during the 4 weeks preceding study entry. Baseline thyroid‐related clinical and treatment information, including disease duration and levothyroxine use, were recorded for all participants.

Participants in the intervention group received Progressive Muscle Relaxation Training (PMRT) based on the Jacobson relaxation method [21]. The protocol involved sequential contraction and relaxation of major muscle groups, including the hands, arms, shoulders, face, chest, abdomen, and lower extremities. Participants initially performed the technique under direct supervision of the researcher to ensure correct execution and received corrective feedback when necessary. Educational materials, including a video demonstration, audio guidance, and illustrated pamphlet, were provided to support home practice. Participants were instructed to perform PMRT twice daily for 20 min over 4 weeks. Adherence was monitored through weekly telephone follow‐ups and Telegram group communication; however, no objective adherence measurement tool was used.

Over the next 4 weeks, participants in the intervention group were instructed to perform PMR technique twice daily (morning and evening), each session lasting 20 min. Patients were also educated to take their levothyroxine medication in the morning on an empty stomach and to refrain from eating for at least 1 h afterward to avoid interfering with medication absorption. Additionally, they were advised not to take iodine and selenium supplements without medical consultation, as these could affect thyroid function. Participants were instructed to maintain a consistent diet throughout the study period and avoid using herbal medications without consulting their physician or informing the researchers. Weekly telephone follow‐ups were conducted to ensure participants adhered to the intervention instructions.

In the control group (B), no information about PMR technique was provided. Participants in the control group continued their routine treatments and were only required to complete the demographic information, Piper Fatigue Scale. They were also monitored weekly through telephone calls, where information about their health status was collected.

At the end of the 4‐week intervention period, the questionnaires were completed again by both groups through telephone contact with researcher number 2, who was blinded to the group allocation. The data collected were then compared between the two groups.

### 2.2 Inclusion Criteria

The inclusion criteria for the study were as follows: the patient should not have communication difficulties, should not have any physical issues preventing participation in exercises, should have full proficiency in Persian, should not have a medical history of neurological or psychiatric disorders, should not be using medications related to these conditions, should voluntarily agree to participate in the study, should be over 18 years old, should not be pregnant (for females), should have clinically confirmed hypothyroidism by a specialist, should not have cognitive impairments, should not have previous experience with PMRT, should own a personal mobile phone or have access to someone with one, and should not have a history of substance abuse.

### 2.3 Exclusion Criteria

Exclusion criteria included unwillingness to continue participation in the study for any reason, development of any debilitating physical condition during the study that would prevent performing exercises, pregnancy during the study period, any medical issues arising during the study, or experiencing a psychological crisis such as grief or divorce during the intervention. Moreover, patients with newly diagnosed hypothyroidism, uncontrolled thyroid disease, secondary hypothyroidism, thyroid malignancy, recent thyroid surgery, or unstable endocrine disorders were excluded from participation.

### 2.4 Data Collection Tools and Methods

#### 2.4.1 Demographic Information Questionnaire

This form included questions about the participant's age, gender, education level, marital status, occupation, disease duration, use of medications for hypothyroidism, and use of other medications.

#### 2.4.2 Piper Fatigue Scale

The Piper Fatigue Scale was designed by Piper et al. in 1998 to assess the perceived fatigue of the patient. This scale evaluates four domains of fatigue: behavioral/severity, affective meaning, sensory, and cognitive/mood dimensions. In this study, raw summed scores were used rather than averaged scores, yielding a total possible score range of 0–220, with higher scores indicating greater fatigue severity. The scale has shown internal consistency with a Cronbach's alpha greater than 0.7 in previous studies.

### 2.5 Beck Depression Inventory‐II (BDI‐II)

The BDI‐II, created by Beck and Steer in 1993, is a 21‐item self‐report scale used to measure the severity of depression. Each item is scored on a scale from 0 to 3. The total score can range from 0 to 63, with higher scores indicating more severe depression. The BDI‐II has been validated in several studies, including a study with a Cronbach's alpha of 0.71 in an Iranian sample.

### 2.6 Sample Size Calculation

The sample size was calculated based on the study by Yoo et al. (2022), using an effect size of 0.5 (medium effect). With a significance level (*α*) of 0.05 and a power (1 − *β*) of 0.80, the sample size for each group was calculated to be 32 participants. Thus, a total of 64 participants were required for the study. A 10% of drop out was considered for this trial and finally the sample size reached to 70 patients.

### 2.7 Ethical Considerations

Prior to the start of the research, written informed consent was obtained from all participants. The consent form explained the study's objectives, methods, potential benefits, and risks. All data collected from the participants were kept confidential and used only for research purposes. The non‐invasive nature of the PMRT technique ensured no physical or psychological harm to the participants. If any adverse effects were observed, the study would be halted immediately. Participant selection was based on the established inclusion and exclusion criteria to ensure fairness and equity.

### 2.8 Statistical Analysis

Data analysis was performed using SPSS software version 22. Continuous variables were presented as mean ± standard deviation (SD), while categorical variables were summarized as frequencies and percentages. Normality of continuous variables was assessed using the Shapiro–Wilk test and visual inspection of histograms. Baseline demographic and clinical characteristics were compared between the intervention and control groups to evaluate the success of randomization. Independent *t*‐tests were used for continuous variables, and Chi‐square or Fisher's exact tests were applied for categorical variables as appropriate.

Within‐group pre‐ and post‐intervention comparisons for fatigue and depression scores were analyzed using paired *t*‐tests. Between‐group differences in change scores were assessed using independent *t*‐tests. Effect sizes were calculated using Cohen's *d*, and 95% confidence intervals (CIs) were reported for mean differences. To further evaluate the independent effect of the intervention while accounting for potential baseline imbalances, adjusted analyses were conducted using multivariable linear regression models. Separate regression models were constructed for fatigue and depression outcomes. The dependent variable in each model was the change score (post‐intervention minus baseline score), and the primary independent variable was group allocation (intervention vs. control). Age, sex, and chronic disease status were entered as covariates because these variables were considered clinically relevant and showed potential baseline imbalance between groups. Adjusted mean differences with 95% confidence intervals and regression coefficients (*β*) were reported.

The analysis was conducted according to a per‐protocol approach, including only participants who completed the study and post‐intervention assessments [22]. No imputation method was applied for missing data because outcome data were available only for participants who completed follow‐up. Statistical significance was considered at a two‐sided *p*‐value < 0.05. The analytical approach was selected to provide both unadjusted and adjusted estimates of intervention effectiveness. Unadjusted analyses allowed direct comparison of pre‐ and post‐intervention changes, whereas multivariable regression models were used to control for clinically important covariates and potential residual confounding despite randomization.

## 3 Results

### 3.1 Demographic Characteristics

Data were analyzed according to the findings of the 68 patients who completed the study. The demographic characteristics of the participants in both the control and intervention groups at baseline were analyzed using Chi‐square tests for categorical variables (gender, marital status, job, residence type, and family history of hypothyroidism) to assess group differences. For continuous variables (age, number of children, weight, and height), independent *t*‐tests were conducted to compare means between the two groups. The results from the demographic analysis are presented in Table 1.

**Table 1:** Demographic analysis.

Variable	Control group	Intervention group	*p*‐value	Effect size (*d*)
Gender				
Female	79.41% [23]	96.97% [24]	0.05	0.24
Male	20.59% [7]	3.03% [1]		
Marital status				
Married	91.18% [25]	78.79% [26]	0.30	0.08
Single	8.82% [3]	18.18% [6]		
Widowed	0% (0)	3.03% [1]		
Job				
Government	26.47% [9]	15.15% [5]	0.19	0.17
Homemaker	64.71% [22]	57.58% [19]		
Self‐employed	8.82% [3]	15.15% [5]		
Unemployed	0% (0)	9.09% [3]		
Residence				
Urban	97.06% [27]	96.97% [24]	1.00	< 0.001
Rural	2.94% [1]	3.03% [1]		
Housing type				
Owned	79.41% [23]	69.70% [28]	0.52	< 0.001
Parental	0% (0)	3.03% [1]		
Rented	20.59% [7]	24.24% [8]		
Addiction history			1.00	N/A
Never	100.00% [34]	100.00% [27]		
Insurance type				
Armed forces	8.82% [3]	6.06% [2]	0.81	< 0.001
No insurance	5.88% [2]	3.03% [1]		
Private	2.94% [1]	0% (0)		
Salamat	20.59% [7]	21.21% [7]		
Social security	61.76% [21]	69.70% [28]		
Chronic disease			0.02	0.26
Yes	76.47% [26]	48.48% [16]		
No	23.53% [8]	51.52% [17]		
Permanent medication			0.36	0.04
Yes	97.06% [27]	90.91% [29]		
No	2.94% [1]	9.09% [3]		
Psychiatric history			1.00	< 0.001
Yes	2.94% [1]	0% (0)		
No	97.06% [27]	100.00% [27]		
Smoking status			0.13	0.18
Current	2.94% [1]	0% (0)		
Former	8.82% [3]	0% (0)		
Never	88.24% [29]	100.00% [27]		
Alcohol consumption			1.00	0.00
Occasional	2.94% [1]	0% (0)		
Never	97.06% [27]	100.00% [27]		
Family history of hypothyroidism			0.61	< 0.001
Yes	61.76% [21]	69.70% [28]		
No	38.24% [13]	30.30% [10]		

### 3.2 Outcome Measures Pre‐ and Post‐Intervention

The outcome measures for both the control and intervention groups before and after the intervention were analyzed using paired *t*‐tests for continuous variables (fatigue and depression). The paired *t*‐test was used to determine whether there was a significant difference between the pre‐ and post‐intervention measures within each group (Tables 2 and 3).

**Table 2:** Control group outcomes.

Outcome	Pre mean ± SD	Post mean ± SD	Change mean ± SD	*p*‐value (paired)	Effect size (*d*)	95% CI for mean change
Fatigue score	81.50 ± 51.18	81.65 ± 51.58	0.15 ± 0.82	0.30	0.18	(−0.50, 0.81)
Depression score	15.50 ± 10.96	15.32 ± 10.81	−0.18 ± 0.72	0.16	−0.25	(−1.08, 0.72)

**Table 3:** Intervention group outcomes.

Outcome	Pre mean ± SD	Post mean ± SD	Change mean ± SD	*p*‐value (paired)	Effect size (*d*)	95% CI for mean change
Fatigue score	88.55 ± 58.01	47.09 ± 51.64	−41.45 ± 51.99	< 0.001	−0.80	(−61.71, −21.19)
Depression score	16.21 ± 8.36	8.12 ± 8.17	−8.09 ± 8.38	< 0.001	−0.97	(−10.58, −5.60)

### 3.3 Between‐Group Comparisons of Changes

**Figure 2:**
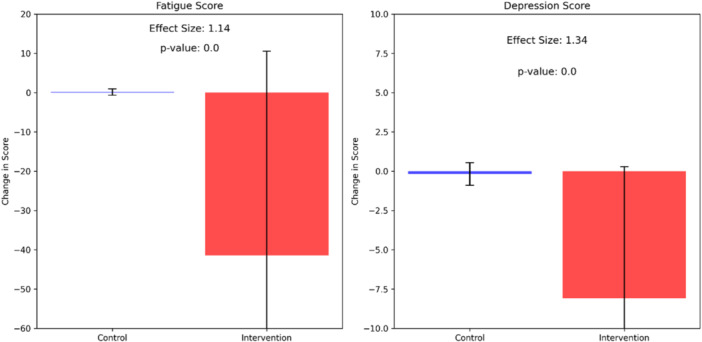
Between‐group comparisons of changes bar chart.

The changes between the control and intervention groups were compared using independent *t*‐tests for continuous variables (fatigue and depression). These tests were used to assess whether the changes observed in the intervention group were significantly different from those in the control group (Table 4) (Figure 2).

**Table 4:** Between‐group comparisons of changes.

Outcome	Control change mean ± SD	Intervention change mean ± SD	*p*‐value	Effect size (*d*)	95% CI for mean difference
Fatigue score	0.15 ± 0.82	−41.45 ± 51.99	< 0.001	1.14	(23.02, 58.87)
Depression score	−0.18 ± 0.72	−8.09 ± 8.38	< 0.001	1.34	(5.87, 7.63)

These results suggest that the intervention had a significant effect on fatigue and depression, with the intervention group showing much larger and statistically significant changes compared to the control group. The control group showed minimal changes in all outcomes.

### 3.4 Adjusted Analysis

After adjusting for sex, chronic disease status, and age, the intervention remained significantly associated with greater reductions in fatigue and depression scores compared with the control group. Baseline covariates were not independently associated with outcome changes (Tables 5 and 6).

**Table 5:** Adjusted between‐group analysis for fatigue and depression outcomes.

Outcome	Adjusted mean difference (intervention vs. control)	95% CI	*p*‐value
Fatigue score	−40.82	−60.51 to −21.12	< 0.001
Depression score	−7.14	−10.29 to −3.99	< 0.001

**Table 6:** Multivariable regression analysis adjusted for baseline covariates.

Covariate	Fatigue outcome (*β*, *p*‐value)	Depression outcome (*β*, *p*‐value)
Intervention group	*β* = −41.36, *p* < 0.001	*β* = −7.26, *p* < 0.001
Sex	*β* = 5.36, *p* = 0.717	*β* = 1.93, *p* = 0.418
Chronic disease	*β* = −6.08, *p* = 0.548	*β* = −0.59, *p* = 0.718
Age	*β* = 0.06, *p* = 0.894	*β* = 0.06, *p* = 0.433

## 4 Discussion

### 4.1 Summary of Main Findings

This randomized controlled study examined the effect of a structured educational and self‐management program on fatigue and depressive symptoms among adults with hypothyroidism. Sixty‐eight patients who completed the study were analyzed. Baseline demographic characteristics were balanced between the control and intervention groups (Table 1), although slightly more women were in the intervention group. Fatigue and depression scores were unchanged in the control group but improved markedly after the intervention: fatigue decreased by approximately 41 points (effect size –0.80) and depression decreased by nearly 8 points (effect size –0.97), whereas the control group showed minimal change (Tables 2, 3, 4). Between‐group comparisons confirmed large effect sizes for both outcomes (fatigue: *d* = 1.14; depression: *d* = 1.34) with confidence intervals excluding zero. These findings indicate that educational and behavioral support can substantially reduce fatigue and depressive symptoms in hypothyroid patients beyond standard care alone.

### 4.2 Comparison With Recent Literature

A growing number of studies published in the past 5 years have explored interventions or associations between hypothyroidism, fatigue, and depression. Several key themes emerge from these studies.

1. **Holistic interventions can reduce fatigue**. A large tele‐yoga randomized controlled trial (*n* = 134) assessed a 6‐month online yoga module for hypothyroid patients. It reported large improvements across all SF‐36 domains (effect sizes ≥ 0.59) and secondary outcomes such as BMI, blood pressure, and the Fatigue Assessment Scale (FAS). FAS scores fell from 42.5 ± 3.2 to 18.1 ± 3.7, while the wait‐list control group's fatigue increased to 39.8 ± 3.8 [18]. This effect size (*η* ^2^ = 0.84) is comparable to the large reduction in fatigue observed in the present study. The intervention also improved energy/vitality and mental health domains.
2. **Pharmacologic treatment improves fatigue but may not fully address depression.** A prospective study of 92 Latino patients receiving levothyroxine showed that median Fatigue Severity Scale (FSS) scores declined from 53 (IQR 47–57) to 36 (16–38) after 6 months (*p* = 0.001). Fatigue frequency dropped from 45.7% to 26.1%. Higher baseline FSS (> 34) and diabetes predicted persistent fatigue [28]. Although levothyroxine improved fatigue, the trial did not evaluate depression; other reports note that thyroid replacement alone may not ameliorate mood disorders [30]. A 10‐week randomized trial of synbiotic supplementation (*n* = 56) found improvements in quality‐of‐life domains (mental health, bodily pain, general health, and wellbeing) but no significant change in depression scores [31]. These studies suggest that pharmacologic or dietary interventions may partially improve fatigue but often leave residual depressive symptoms, supporting the need for complementary psychosocial strategies.
3. **High prevalence of depression among hypothyroid patients and the role of fatigue as a risk factor.** A cross‐sectional survey from Saudi Arabia (*n* = 100) found that 80% of hypothyroid patients met criteria for depression (35% mild, 26% moderate, and 19% moderate/severe). Depression was strongly associated with fatigue (*p* < 0.001) and other symptoms, but in multivariate analysis only fatigue remained significant (OR = 15.2) [26]. Likewise, a Senegalese study (*n* = 40) reported that anxiety and depressive symptoms were prevalent (67% and 65%, respectively) and associated with elevated TSH (> 4.5 mIU L^−1^) and poor treatment adherence [23]. These findings underscore that fatigue is both a symptom and a predictor of depression, and that endocrine imbalance and poor adherence contribute to psychological distress [32]. In our study, participants with a family history of hypothyroidism or chronic disease reported higher baseline fatigue and depression, and the intervention targeted coping skills and adherence, which may explain the significant improvements.
4. **Pathophysiological links between thyroid dysfunction and mood disorders.** A narrative review noted that undiagnosed or undertreated hypothyroidism increases the risk of depression, and that elevated TSH, antithyroglobulin and thyroid‐peroxidase antibodies are linked to depression and suicidality. Depression and hypothyroidism may have a bidirectional relationship through the hypothalamic–pituitary–thyroid axis. Previous studies have suggested that major depression can blunt the thyroid‐stimulating hormone (TSH) response to thyrotropin‐releasing hormone (TRH) and reduce the nocturnal TSH surge; however, these neuroendocrine mechanisms were not directly evaluated in the present study [33]. Mendelian‐randomization studies indicate that genetically predicted hypothyroidism is associated with major depression and that major depression significantly increases the risk of hypothyroidism [29, 33]. These mechanistic insights highlight the importance of addressing both endocrine and psychological factors. Our intervention included stress management and education about thyroid disease, which may have influenced psychoneuroendocrine pathways, consistent with the improvements observed.

### 4.3 Clinical Implications and Implementation

#### 4.3.1 Integrating Behavioral Interventions Into Endocrine Care

The present study and the tele‐yoga trial demonstrate that structured behavioral interventions can substantially reduce fatigue and depressive symptoms in hypothyroid patients. These programs may improve outcomes by enhancing self‐management, promoting regular physical activity, enhancing coping skills, and providing social support, thereby modulating the hypothalamic–pituitary–thyroid axis and reducing stress [25]. Clinicians should consider integrating evidence‐based behavioral or mind–body programs such as education, cognitive‐behavioral therapy, yoga, mindfulness, or NET into routine hypothyroidism management, particularly for patients with persistent fatigue or depressive symptoms despite adequate hormone replacement.

Implementation can be facilitated through group sessions or telehealth platforms. The tele‐yoga trial achieved high adherence (≈83%) and low attrition, demonstrating the feasibility of remote delivery. Educational components should include information on thyroid physiology, medication adherence, lifestyle modifications (diet, exercise, and sleep), stress management, and recognition of depressive symptoms [24]. Collaborative care models involving endocrinologists, mental‐health professionals, and physiotherapists can provide comprehensive support [18].

#### 4.3.2 Screening and Monitoring

Given the high prevalence of depression (60%–80%) in cross‐sectional studies, routine screening for fatigue and depressive symptoms should be incorporated into hypothyroidism clinics [27]. Validated instruments such as the Fatigue Severity Scale, Patient Health Questionnaire‐9, or Hospital Anxiety and Depression Scale can be administered at baseline and follow‐up. Abnormal scores warrant referral to mental‐health services and consideration of adjunctive psychosocial interventions.

Thyroid function should continue to be monitored during behavioral programs. While levothyroxine remains the standard of care, some patients experience persistent symptoms despite normal TSH. Research into combination therapy with slow‐release liothyronine is ongoing, but robust outcomes are not yet available. Emerging evidence from MR studies suggests that depression may causally influence thyroid function; hence, addressing depression might improve endocrine outcomes [33].

### 4.4 Adjusted Analyses

Adjusted analyses showed that the intervention remained significantly associated with reductions in fatigue and depression scores after controlling for important baseline variables, including sex, age, and chronic disease status. These findings indicate that the observed improvements were not explained by demographic or clinical differences between groups. The persistence of significant treatment effects following multivariable adjustment strengthens the internal validity of the study and supports the potential clinical effectiveness of the intervention in improving psychological and fatigue‐related outcomes among patients with hypothyroidism.

### 4.5 Baseline Imbalance and Potential Residual Confounding

A baseline imbalance was observed between the study groups in chronic disease prevalence and sex distribution. The control group had a higher proportion of participants with chronic diseases, while the intervention group included a greater proportion of female participants. These differences may have influenced fatigue and depression outcomes and therefore represent potential sources of residual confounding despite randomization. To minimize this effect, adjusted multivariable regression analyses were performed controlling for age, sex, and chronic disease status, and the intervention effect remained statistically significant after adjustment. Nevertheless, residual confounding cannot be completely excluded because of the relatively small sample size and unequal baseline distribution of some clinical characteristics. These findings should therefore be interpreted with caution, and future studies with larger samples and stratified randomization are recommended.

## 5 Limitations and Future Research

This study has limitations. The sample size was modest and predominantly female, which may limit generalizability. Blinding was not feasible, which may introduce expectancy effects. The control group received standard care but no attention placebo; thus, non‐specific effects could contribute to the observed improvements. Follow‐up was limited; long‐term sustainability of benefits remains uncertain. Moreover, the intervention group received greater attention, monitoring, and behavioral support than controls, increasing potential expectancy and attention biases and limiting the ability to attribute observed effects specifically to PMR alone. A further limitation was the absence of post‐intervention thyroid function assessments, preventing evaluation of whether symptom improvements were accompanied by objective hormonal or biochemical changes. A limitation of this study was the significant baseline difference in chronic disease prevalence between groups, which may have influenced fatigue and depression outcomes despite adjusted analyses remaining statistically significant.

Future research should include larger multi‐center trials with longer follow‐up to confirm efficacy and explore cost‐effectiveness. Comparative studies could test different behavioral modalities (yoga vs. cognitive‐behavioral therapy vs. NET) and examine which components drive improvement. Combining behavioral interventions with pharmacologic optimization or synbiotic supplementation may yield synergistic benefits. Mechanistic studies evaluating neuroendocrine and immune biomarkers could clarify how psychosocial interventions modulate the hypothalamic–pituitary–thyroid axis.

## 6 Conclusion

The present randomized study adds to the growing evidence that combined educational and behavioral interventions may improve fatigue and depressive symptoms in patients with hypothyroidism. The intervention package included Progressive Muscle Relaxation (PMR), patient education, adherence counseling, weekly telephone follow‐up, and messaging support, and was associated with significant improvements compared with routine care alone. These findings suggest that supportive non‐pharmacological strategies targeting psychological well‐being and treatment adherence may help reduce persistent symptoms and improve quality of life in hypothyroid patients. Incorporating psychological assessment and multidisciplinary supportive care into routine endocrine management may further enhance patient outcomes. However, because several supportive components were delivered simultaneously, the observed benefits cannot be attributed solely to PMR. Further research is needed to determine the independent effects and optimal implementation of these interventions.

## Author Contributions

**Mehdi Safarabadi:** conceptualization, investigation, methodology, supervision. **Marzieh Mousivand:** conceptualization, investigation, data curation. **Hooman Mohammad Talebi:** supervision, software, formal analysis, writing – review and editing, writing – original draft. **Hossein Poorcheraghi:** investigation, methodology. **Sara Mohammadi Farsani:** conceptualization, investigation, methodology.

## Funding

The authors have nothing to report.

## Ethics Statement

This study was conducted in accordance with the Declaration of Helsinki. An informed consent was obtained from the patients prior to the intervention. The ethical approval code was obtained from the Khomein University of Medical Sciences (IR.KHOMEIN.REC.1404.009). Trial Registration code was obtained from Iranian Registry of Clinical Trials (IRCT20250605066079N1) on 2025‐07‐04.

## Conflicts of Interest

The authors declare no conflicts of interest.

## Transparency Statement

The lead/corresponding author (Sara Mohammadi Farsani) affirms that this manuscript is an honest, accurate, and transparent account of the study being reported; that no important aspects of the study have been omitted; and that any discrepancies from the study as planned (and, if relevant, registered) have been explained.

## Data Availability

The data that support the findings of this study are available from the corresponding author upon reasonable request.
